# External Drive to Inhibitory Cells Induces Alternating Episodes of High- and Low-Amplitude Oscillations

**DOI:** 10.1371/journal.pcbi.1002666

**Published:** 2012-08-30

**Authors:** Oscar J. Avella Gonzalez, Karlijn I. van Aerde, Ronald A. J. van Elburg, Simon-Shlomo Poil, Huibert D. Mansvelder, Klaus Linkenkaer-Hansen, Jaap van Pelt, Arjen van Ooyen

**Affiliations:** 1 Department of Integrative Neurophysiology, Center for Neurogenomics and Cognitive Research, VU University Amsterdam, Amsterdam, The Netherlands; 2 Institute of Artificial Intelligence, Faculty of Mathematics and Natural Sciences, University of Groningen, Bernoulliborg, Groningen, The Netherlands; Indiana University, United States of America

## Abstract

Electrical oscillations in neuronal network activity are ubiquitous in the brain and have been associated with cognition and behavior. Intriguingly, the amplitude of ongoing oscillations, such as measured in EEG recordings, fluctuates irregularly, with episodes of high amplitude alternating with episodes of low amplitude. Despite the widespread occurrence of amplitude fluctuations in many frequency bands and brain regions, the mechanisms by which they are generated are poorly understood. Here, we show that irregular transitions between sub-second episodes of high- and low-amplitude oscillations in the alpha/beta frequency band occur in a generic neuronal network model consisting of interconnected inhibitory and excitatory cells that are externally driven by sustained cholinergic input and trains of action potentials that activate excitatory synapses. In the model, we identify the action potential drive onto inhibitory cells, which represents input from other brain areas and is shown to desynchronize network activity, to be crucial for the emergence of amplitude fluctuations. We show that the duration distributions of high-amplitude episodes in the model match those observed in rat prefrontal cortex for oscillations induced by the cholinergic agonist carbachol. Furthermore, the mean duration of high-amplitude episodes varies in a bell-shaped manner with carbachol concentration, just as in mouse hippocampus. Our results suggest that amplitude fluctuations are a general property of oscillatory neuronal networks that can arise through background input from areas external to the network.

## Author Summary

Rhythmic changes in electrical activity are observed throughout the brain, and arise as a result of reciprocal interactions between excitatory and inhibitory neurons. Synchronized activity of a large number of neurons gives rise to macroscopic oscillations in electrical activity, which can be measured in EEG recordings and are thought to have a key role in learning and memory. Interestingly, the amplitude of ongoing oscillations fluctuates irregularly, with high-amplitude episodes alternating with low-amplitude episodes. Although these amplitude fluctuations occur in many brain regions, the mechanisms by which they are generated are still poorly known. To get insight into potential mechanisms, we investigated whether such fluctuations occur in a computational model of a neuronal network. We show that the model generates amplitude fluctuations that are similar to those observed in experimental data and that external input from other brain areas to the inhibitory cells of the network is essential for their generation. This input can disrupt the synchrony of activity, causing transitions between episodes of high synchrony (high oscillation amplitudes) and episodes of low synchrony (low oscillation amplitudes). Episodes of high synchrony are relevant for brain function because they provide favorable conditions for learning.

## Introduction

Oscillations in electrical activity are a characteristic feature of many brain networks, including the hippocampus [1], [2], prefrontal cortex [3], visual cortex [4], [5] and auditory cortex [6], and arise as a result of interacting excitatory and inhibitory populations of cells [7], [8], [9]. Network oscillations occur at many frequencies, ranging from fast gamma (40–80 Hz) to ultra-slow delta (0.1–1 Hz) [10], [11]. Oscillations are linked with cognitive and behavioral functions, including attention [12], [13], [14], learning [10], [15], working memory [16], [17], [18] and memory consolidation [19], and show abnormalities in neurological disorders such as autism, schizophrenia and attention-deficit hyperactivity disorder (ADHD) [16], [20], [21], [22], [23], [24].

Intriguingly, in EEG and MEG recordings the amplitude of ongoing oscillations fluctuates irregularly, with high-amplitude episodes (HAEs) alternating with low-amplitude episodes (LAEs). These amplitude fluctuations, which are generated locally and are different from the well understood thalamocortical spindles [25], [26], [27], have been observed in the intact brain [28], [29], [30] as well as in cortical slices [3], [31], [32] and occur in many frequency bands, including theta (4–6 Hz) [33], alpha (8–13 Hz) [34], beta (14–30 Hz) [32], and gamma (25–80 Hz) [35] bands. Amplitude fluctuations are present in ongoing brain activity during rest [36], while sustained increases in oscillation amplitude are associated with the performance of memory-related tasks [37], [38]. Alterations in the temporal structure of amplitude fluctuations have been observed in Alzheimer's disease [39] and ADHD [21].

Despite the widespread occurrence of amplitude fluctuations, and some theoretical efforts to understand them [40], [41], [42], the mechanisms by which they are generated are poorly known. To get insight into potential mechanisms, we investigated whether such fluctuations may also occur in a computational model of a generic neuronal network consisting of interconnected inhibitory and excitatory cells. We found that the model generated oscillations in the alpha/beta-frequency band, with distributions of HAE durations similar to those observed in experimental data of carbachol-induced oscillations in prefrontal cortex (PFC) slices [3], [32]. Moreover, the relationship between HAE duration and cholinergic drive in the model was similar to that in mouse hippocampus *in vitro* [42].

Our results suggest that fluctuations in oscillation amplitude can arise as a result of a temporarily decrease in firing synchrony caused by the interference between the ongoing network-generated oscillatory activity and input to the inhibitory population originating from areas external to the network.

## Methods

### Experimental data on amplitude fluctuations in PFC and hippocampus

**Figure 1:**
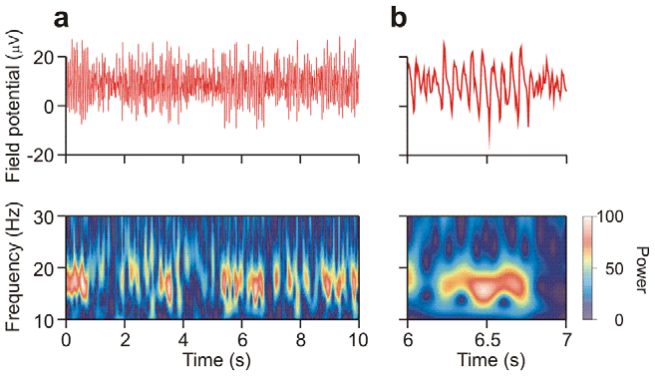
Amplitude fluctuations in carbachol-induced oscillations recorded in the infralimbic region of the PFC. (a) Extracellular field potential (top) at one of the 64 electrodes of a multi-electrode array, and wavelet transform (bottom). Episodes of high power are observed to alternate with episodes of low power. Color indicates power of oscillations. (b) Close up of the activity in (a).

**Figure 2:**
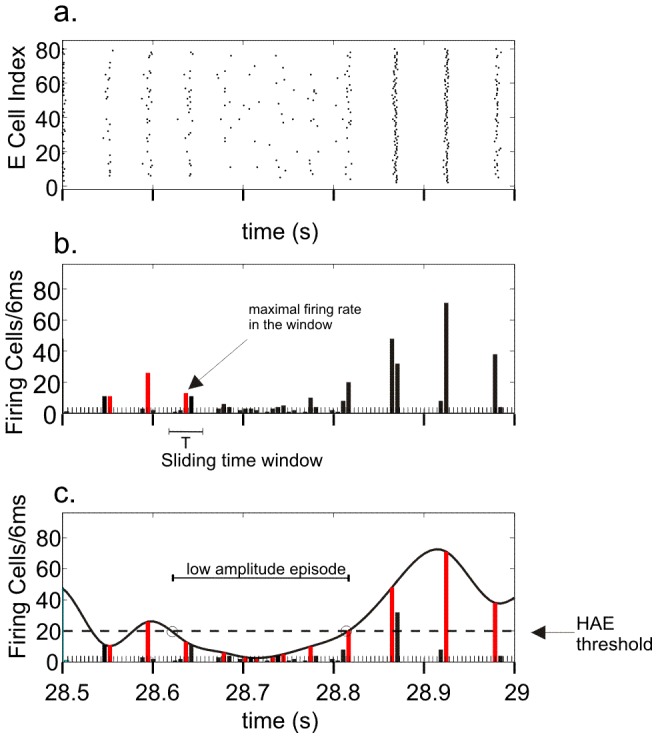
Quantification of high-amplitude episodes (HAEs) and low-amplitude episodes (LAEs) in network oscillations. (a) Raster diagram showing the firing times (indicated by dots) of the excitatory cells. (b) Corresponding firing-rate histogram. The maximal firing rate (red bar) per oscillation period *T* is successively determined by using a sliding time window of length *T*. The time axis is discretized into bins of 6 ms. (c) A spline polynomial is interpolated through the maximal firing rates (red bars) per oscillation period. Time intervals during which the curve exceeds the HAE threshold (dashed line) are considered HAEs, otherwise LAEs. (See further Methods.)

In our earlier work [32], [40], [41], [42], we observed amplitude fluctuations in oscillations in EEG and MEG recordings from human subjects, in *in vivo* recordings of local field potentials in the prefrontal cortex (PFC) of freely moving rats, as well as in *in vitro* recordings of local field potentials (Fig. 1) in acute slices of the rat PFC. The durations of high-amplitude episodes (HAEs) were quantified for multiple recordings of 200 seconds in 25 recordings from medial prefrontal cortex (mPFC) slices [32]. Data on the effect of increasing concentrations of carbachol on HAEs in mouse hippocampus *in vitro* was taken from [42] (Fig. 2 therein).

### Model cells

Following [7] and [43], we built a model neuronal network in NEURON [44], consisting of 80 excitatory cells and 20 inhibitory cells (Fig. S1), reflecting the ratio of excitatory to inhibitory cell numbers found in most cortical areas [45]. Cells were defined as one-compartment, conductance-based models, with a length and diameter of 20 µm, and contained the Hodgkin-Huxley Na^+^ and K^+^ channels, responsible for action potential generation, as well as leakage channels. The change in membrane potential *V* (in mV) was given by
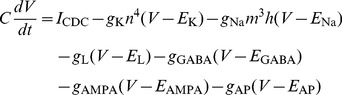
(1)with time *t* in ms; 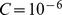 F/cm^2^ the membrane capacitance; 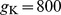 pS/µm^2^ and 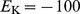 mV the conductance and reversal potential of the K^+^ channels; 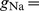
1000 pS/µm^2^ and 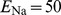 mV the conductance and reversal potential of the Na^+^ channels; and 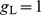 pS/µm^2^ and 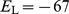 mV the conductance and reversal potential of the leakage channels. Each cell received synaptic input from other cells in the network, with 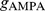 and 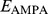 the synaptic conductance and reversal potential of the AMPA channels; and 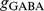 and 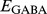 the synaptic conductance and reversal potential of the GABA_A_ channels (for parameter values, see Model network). In addition, each cell received two kinds of external input: a constant depolarizing current 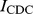 and a train of external action potentials impinging onto an excitatory synapse, with synaptic conductance 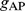 and reversal potential 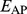 (for parameter values, see External drive). All parameter values were as in [46].

The dynamics of the gating variables *n*, *m* and *h* (in general denoted by *z*) of the ion channels were given by
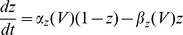
(2)with 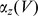 and 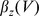 the voltage-dependent opening and closing rate constants. For the *n*, *m* and *h* variables, these functions were [46]:
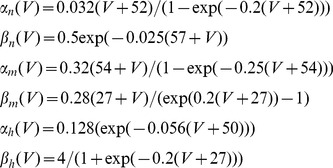
(3)


### Model network

The connectivity structure of the network was created by giving each cell a probability to connect to any other cell. Excitatory (E) cells connected to inhibitory (I) and E cells with probabilities 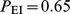 and 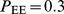, respectively; while I cells connected to E and I cells with probabilities 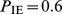 and 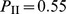, respectively. A connection consisted of a single synapse with a synaptic conductance as described below. The connectivity structure was chosen on the basis of the following considerations. First, the use of connection probabilities prevents unrealistic all-to-all connectivity [47]. Second, the connection probabilities should be high enough to create a globally connected network rather than a number of isolated subnetworks. Third, and most importantly, the oscillations should be generated by a so-called PING (Pyramidal Interneuron Network Gamma) mechanism [48]. In this mechanism, which underlies the generation of most oscillations in the brain, the E cells (pyramidal cells) activate the I cells (interneurons), which in turn suppress the E cells. The PING mechanism depends on strong connectivity from E to I cells, strong connectivity from I to E cells, and, to promote synchronous firing, connectivity from I to I cells [48].

For both excitatory and inhibitory synapses, the time course of the synaptic conductance was given by a mono-exponential function. The synaptic delay for both type of synapses is 1 ms [49]. Synaptic excitation was mediated by AMPA synapses with a conductance 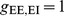 pS/µm^2^, reversal potential 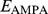 = 0 mV and decay time constant 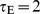 ms [7], [8], [50]. Synaptic inhibition was mediated by 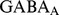 synapses with conductances 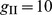 pS/µm^2^ and 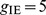 pS/µm^2^, reversal potential 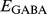 = −80 mV, and decay time constant 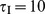 ms, unless stated otherwise. These parameters were as in [46], [51]. The decay time constant of the inhibitory conductance (i.e., the IPSC decay constant; 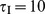 ms) tuned the network oscillations into the alpha/beta frequency band, with oscillations of about 18 Hz, in the middle of the range of frequencies reported for PFC slices [3], [32], [52].

### External drive

As in [7], each cell received two kinds of external input (see eqn. 1): a constant depolarizing current 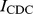, representing cholinergic input necessary to induce oscillations [9], [53], and a train of external action potentials (AP), representing background input from other brain areas [54].

Cholinergic input has been shown to cause a sustained depolarizing response [55], which, as we do here, can be mimicked by applying a non-specific, depolarizing current to the cells [9]. The amplitude of 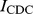 varied among cells and was randomly drawn from a uniform distribution in the intervals 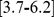 pA for the inhibitory population and 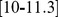 pA for the excitatory population; these currents alone were not strong enough to trigger action potentials.

The train of external action potentials (AP) is characterized by its randomness and mean firing frequency. The randomness (*rand*) is denoted as a fixed number in the interval [0, 1], with 0 indicating no randomness and 1 indicating full randomness of the Poisson-distributed spike train. The mean firing frequency is equal to 1/*isi*, where *isi* is the mean interspike interval. The firing time of the first external spike was set at 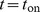; the firing times of all subsequent spikes were computed by
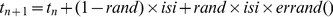
(4)where 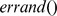 is a random number drawn from a negative exponential distribution with a mean of 1. Unless stated otherwise, the first spike was generated at 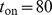 ms. All cells received external spike trains independently from each other. External action potentials activated an excitatory synapse with conductance 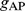 = 2.6 pS/µm^2^, reversal potential 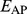 = 0 mV and decay time constant 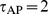 ms.

### Analyzing network activity

Network activity was analyzed separately for the excitatory and the inhibitory population. To describe the time-domain of network activity in the model, we constructed firing-rate histograms by counting spikes in time bins of 6 ms. This bin size relates to a sample frequency of about 167 Hz, about 5 times higher than the fastest frequency (30 Hz) in our simulations. Because this bin size practically eliminated the occurrence of more than one spike per time bin per cell, the number of spikes was equal to the number of active cells per time bin.

To analyze the time-frequency domain of network activity in the model, we performed a wavelet analysis using the Torrence algorithm [56], implemented in MatLab and with the 6 ms-binned firing rate histogram as input. A standard Morlet function was used with a frequency range 0.01–70 Hz and 0.1 Hz scaling windows. A wavelet analysis reveals how the power (amplitude) of oscillations varies over time.

To quantify the amplitude modulation of the oscillations in more detail, we identified the maximal firing rates in all successive periods of the oscillation (Fig. 2). To this end, first a rough estimate of the oscillation period *T* was obtained by determining the average time duration between the time bins for which the firing rates exceeded the mean firing rate. Next, a sliding time window with length *T* was used to search for the time bins with the highest firing rate per period. The procedure started by finding the first time bin *t*(1) with the highest amplitude, which marked the peak firing rate in the first period of the oscillation. The sliding time window was centered around *t*(1), thus covering the range [*t*(1)−*T*/2, *t*(1)+*T*/2]. Next, the window was shifted to [*t*(1)+*T*/2, *t*(1)+*T*/2+*T*] to find time bin *t*(2) with the maximal firing rate in the second period of the oscillation. Subsequently, the time window was shifted to [*t*(2)+*T*/2, *t*(2)+*T*/2+*T*] to find time bin *t*(3), and so on. Thus, iteratively, time bin *t*(*i*+1) with maximal firing rate is searched for within the time window [*t*(*i*)+*T*/2, *t*(*i*)+*T*/2+*T*]. Because of the alignment (and *T*/2 shift) of the sliding window with the previously found peak bin, the procedure is insensitive to small momentary variations in the periodicity of the oscillation.

Then, a smooth curve through the maximal firing rates was obtained by interpolating a third-order spline polynomial. This interpolated curve was used to quantify high-amplitude episodes (HAEs) and low-amplitude episodes (LAEs). When for a given time interval the interpolated curve exceeded a given threshold, the interval was considered a HAE, otherwise a LAE. This HAE threshold was set at 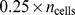, where 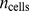 is the total number of cells in the excitatory or the inhibitory population. In other words, a HAE was defined as an episode in which at least 25% of a neuronal subpopulation fired synchronously with a precision of 6 ms (the size of the time bins).

To compare our model outcomes with experimental data on amplitude fluctuations in PFC and hippocampus, we took the population firing-rate histograms as being representative of local field potential. Although determining the precise relation between neuronal firing and field potential is still a topic of ongoing research and would require extensive knowledge about the cellular and extracellular conductive environment [57], both modeling studies [58] and experimental findings [59] appear to support a direct relationship between local field potential and population firing rate.

## Results

To get insight into potential mechanisms underlying the amplitude fluctuations observed in ongoing oscillations in electrical activity [29], [30], we investigated whether such fluctuations may also occur in a computational model of a generic neuronal network consisting of interconnected inhibitory and excitatory cells that are externally driven by sustained cholinergic input and excitatory synaptic input.

### Fluctuations in oscillation amplitude

**Figure 3:**
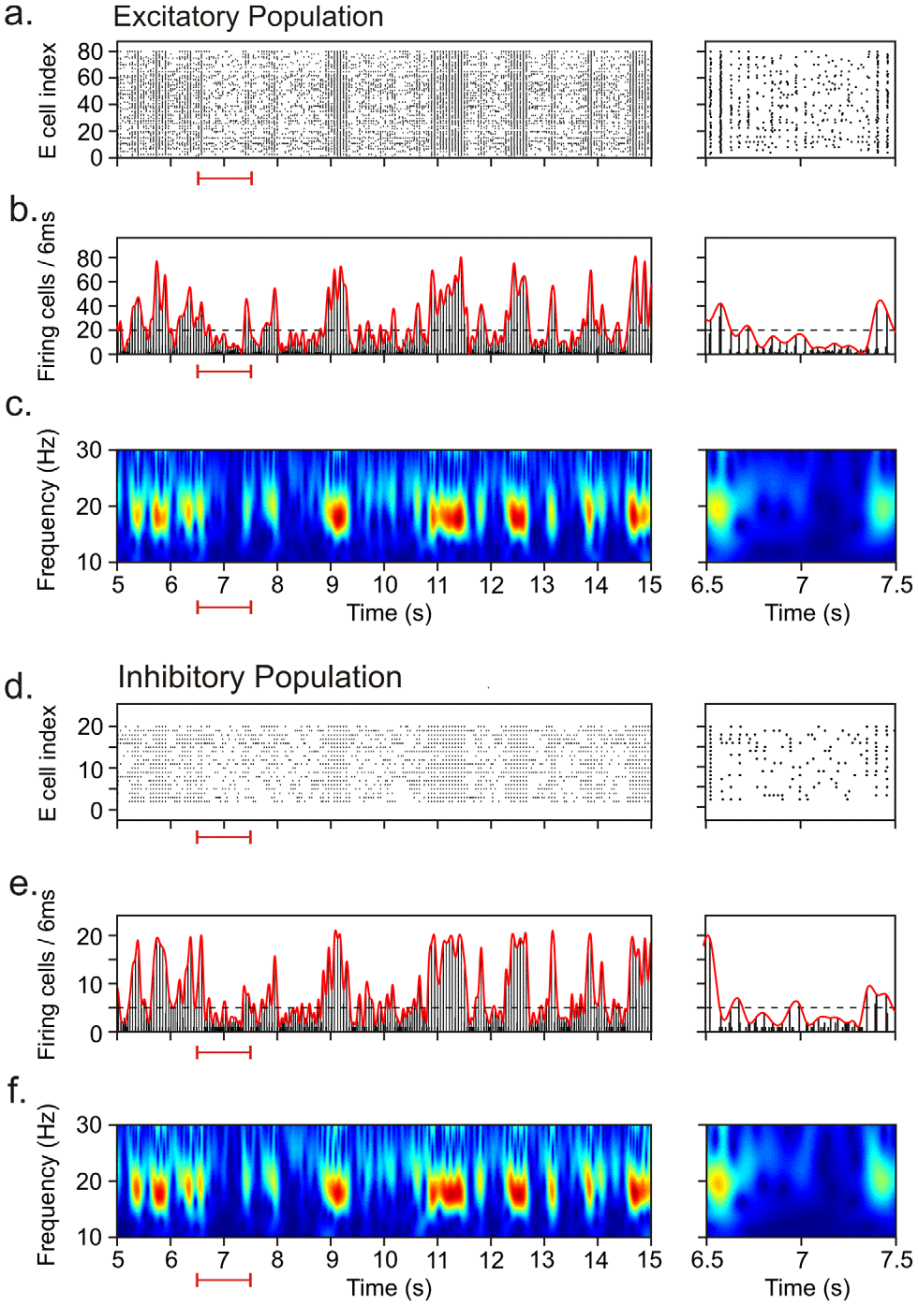
Amplitude fluctuations in oscillations generated in the neuronal network model, with high-amplitude episodes (HAEs) alternating with low-amplitude episodes (LAEs). The dynamics of alternating HAEs and LAEs occurred in both the excitatory and the inhibitory population. Representative activity is shown separately for the excitatory and the inhibitory population as raster diagrams of cell firing (a, d), firing-rate histograms with interpolated spline polynomials (b, e; red lines on top of histograms), and wavelet transforms of the firing-rate histograms (c, f). The episodes in which the interpolated polynomials (b, e) exceeded the dashed horizontal line (the HAE threshold) are considered HAEs, otherwise LAEs. The excitatory and the inhibitory population exhibited similar dynamics with respect to HAE-LAE alternations. The panels on the right are zoomed-in intervals indicated by the red horizontal lines below the x-axes. The external input to the inhibitory cells consisted of a constant depolarizing current (CDC) and a train of action potentials (AP) activating an excitatory synapse. The excitatory cells received external input only in the form of CDC input.

The neuronal network model was found to generate strong fluctuations in oscillation amplitude, with high-amplitude episodes (HAEs) alternating with low-amplitude episodes (LAEs). Fig. 3 shows an arbitrary 10 seconds interval of network activity in both the excitatory population (Fig. 3a–c) and the inhibitory population (Fig. 3d–f). In this example, the inhibitory cells received external input in the form of a constant depolarizing current (CDC) and a train of action potentials (AP) that activate an excitatory synapse. The excitatory cells received external input only in the form of CDC input. The CDC input represents cholinergic input, and the AP input reflects background synaptic input from areas external to the network. As can be seen in Fig. 3, both the excitatory and the inhibitory population exhibited HAEs and LAEs, and in most cases the transitions between HAEs and LAEs occurred around the same time in both populations.

**Figure 4:**
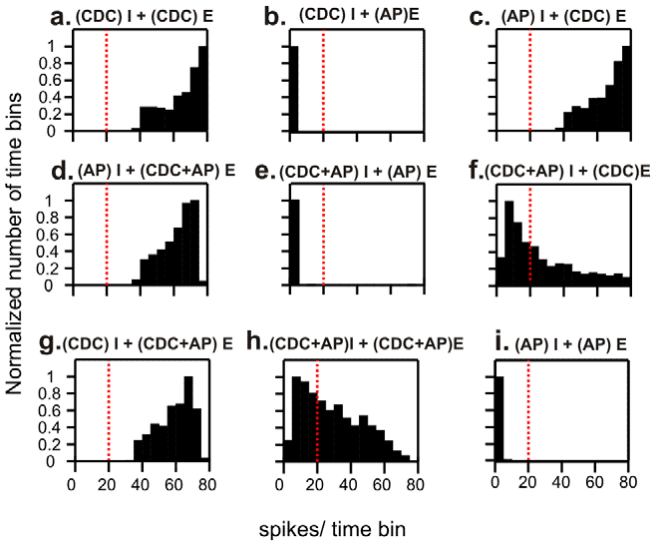
Alternations between episodes of high- and low-amplitude oscillations occurred only when both the inhibitory (I) and the excitatory (E) cells received an external constant depolarizing current (CDC) and at least the inhibitory cells received a train of external action potentials (AP) activating an excitatory synapse. Each panel shows the distribution of oscillation amplitudes (in terms of number of spikes per time bin) in the excitatory population for the nine different combinations of external input to the network. Thus, alternations between high-amplitude episodes (HAEs) and low-amplitude episodes (LAEs) occurred only when the distribution contained oscillation amplitudes at both sides of the HAE threshold (the dotted vertical red line; scenarios f and h). The distributions were normalized by dividing the number of time bins by the maximal number of time bins in the distribution.

To explore further what stimulus conditions induced fluctuations in oscillation amplitude, we simulated networks with all possible combinations of external spike (AP) and current (CDC) input to the excitatory and inhibitory populations (Fig. 4). The interacting excitatory and inhibitory cell populations were found to produce strong oscillations (at around 18 Hz) only in the presence of CDC input to the excitatory population, in line with experimental data showing the dependence of oscillations on depolarizing cholinergic drive to pyramidal cells [2], [32] Setting a threshold for defining a high oscillation amplitude (HAE threshold; see Methods), we found that depending on the input conditions, oscillation amplitudes remained supra-threshold (Figs. 4a, c, d, g; Fig. S2), sub-threshold (Figs. 4b, e, i), or adopted values both above and below threshold (Figs. 4f, h). Thus, pronounced alternations between high- and low-amplitude episodes occurred only with CDC+AP input to the inhibitory cells and CDC input (with or without AP input) to the excitatory cells (Figs. 4f, h). HAE-LAE alternations were also not observed in the scenario of Fig. 4d if, to compensate for the lack of CDC input to the inhibitory cells, the AP frequency or the strength of the excitatory synapses activated by the APs was scaled up. In that situation, the AP input became so strong that the inhibitory cells responded mainly to the random AP input rather than to the input from within the network, resulting in asynchronous activity and consequently the absence of network oscillations. Likewise, no HAE-LAE alternations occurred in the scenario of Fig. 4g if, to compensate for the lack of AP input to the inhibitory cells, the CDC input was increased, indicating that the discontinuous action potential input rather than the continuous current input drives the transitions from HAEs to LAEs (see further below). Thus, the *minimal stimulation condition* for producing HAE-LAE alternations is CDC input applied to both populations and AP input applied to the inhibitory population. Unless mentioned otherwise, the minimal stimulation protocol was used for all other simulations.

### AP randomness and frequency influence durations of HAEs and LAEs

The network activity in Fig. 3 was generated using a completely random AP input (*rand* = 1) with a mean firing frequency of 11.11 Hz (*isi* = 90 ms). To study how the characteristics of the AP input affected fluctuations in oscillation amplitude, we systematically varied AP randomness and AP frequency. Alternating periods of high- and low-amplitude oscillations were found to occur for a wide range of AP randomness (0–1) and AP frequency (4–35 Hz) (Fig. S3).

**Figure 5:**
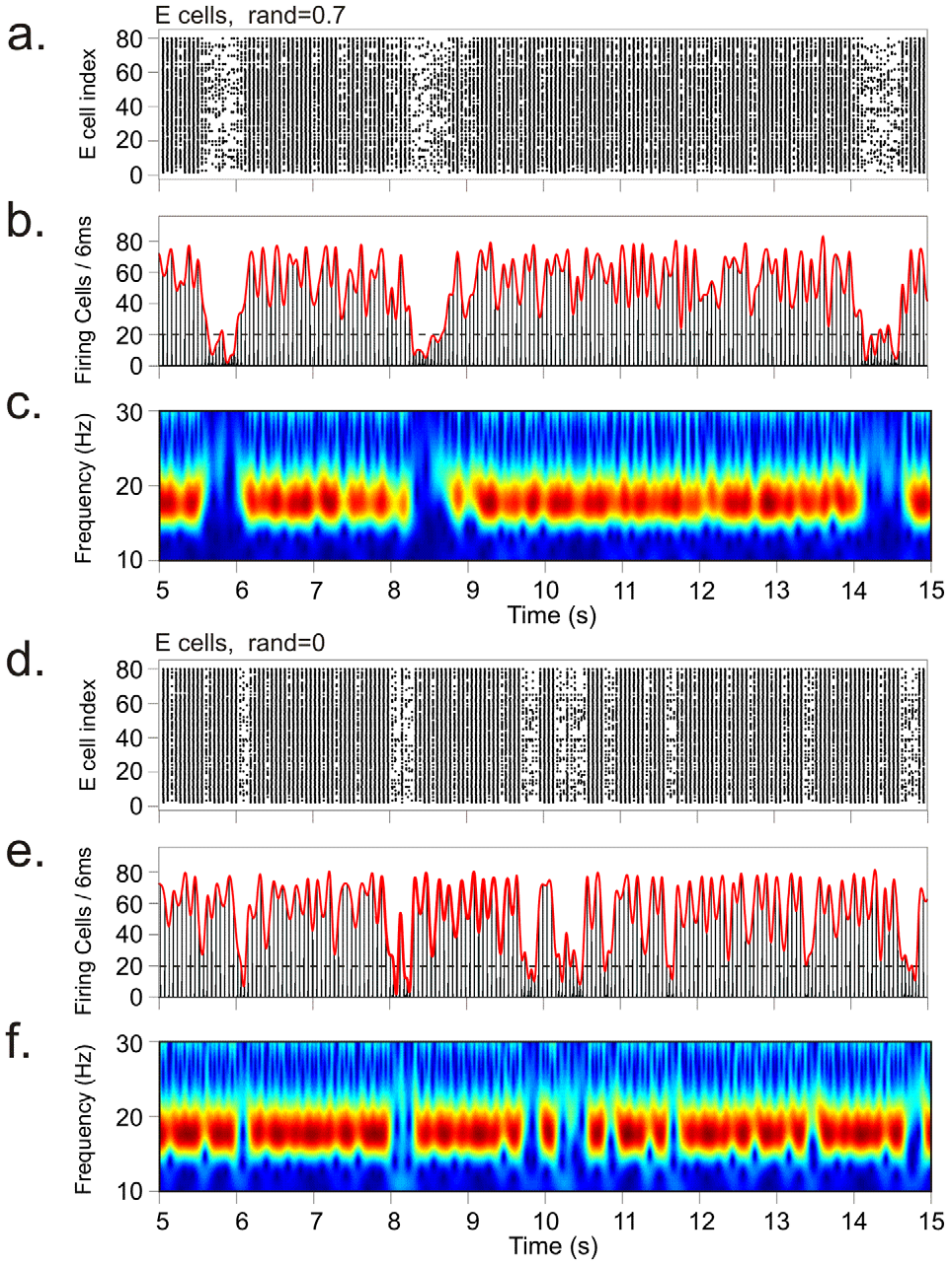
Alternating episodes of high- and low-amplitude oscillations for two different values of AP randomness. Raster diagrams of cell firing (a, d), firing-rate histograms with interpolated spline polynomials (b, e) and wavelet transform of the firing-rate histograms (c, f) for the excitatory population for AP randomness 0.7 (a–c) and 0 (d–f) in the minimal stimulation protocol. For rand = 0, APs were simultaneously delivered to all I cells at regular intervals of 90 ms.

**Figure 6:**
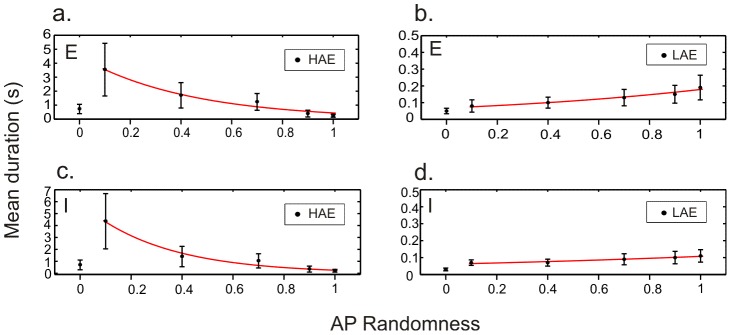
The more random the AP train, the shorter the mean HAE duration and the longer the mean LAE duration. Mean HAE and LAE durations (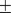
SEM) in the excitatory population (a, b) and the inhibitory population (c, d) for different values of AP randomness. Red lines, exponential fits.

To investigate the effect of AP randomness, we fixed AP frequency (*isi* = 90 ms) and varied AP randomness between completely regular (*rand* = 0) and fully random (*rand* = 1). Examples of network activity for two different values of AP randomness (0.7 and 0) are shown in Fig. 5. As can be seen, even with a completely regular AP train (*rand* = 0), HAE-LAE alternations occurred (Fig. 5d–f); so, importantly, AP randomness is not essential. When AP randomness was increased, the mean LAE duration increased and the mean HAE duration decreased, both in the excitatory population (Fig. 6a, b) and in the inhibitory population (Fig. 6c, d). (The trend is broken for *rand* = 0.) Fig. S4 shows the distributions of HAE and LAE durations for different values of AP randomness.

In the default scenario for a completely regular AP train (*rand* = 0), all inhibitory cells received the external spikes at the same time, with the onset of spiking in all AP trains at 80 ms and AP frequency 11.11 Hz (the frequency of the ongoing oscillation is around 18 Hz). We also studied a second scenario in which the onset of spiking was fully randomized between 0 and 80 ms, producing AP trains that were out-of-phase among each other. Both scenarios produced HAE-LAE alternations (Fig. S5).

**Figure 7:**
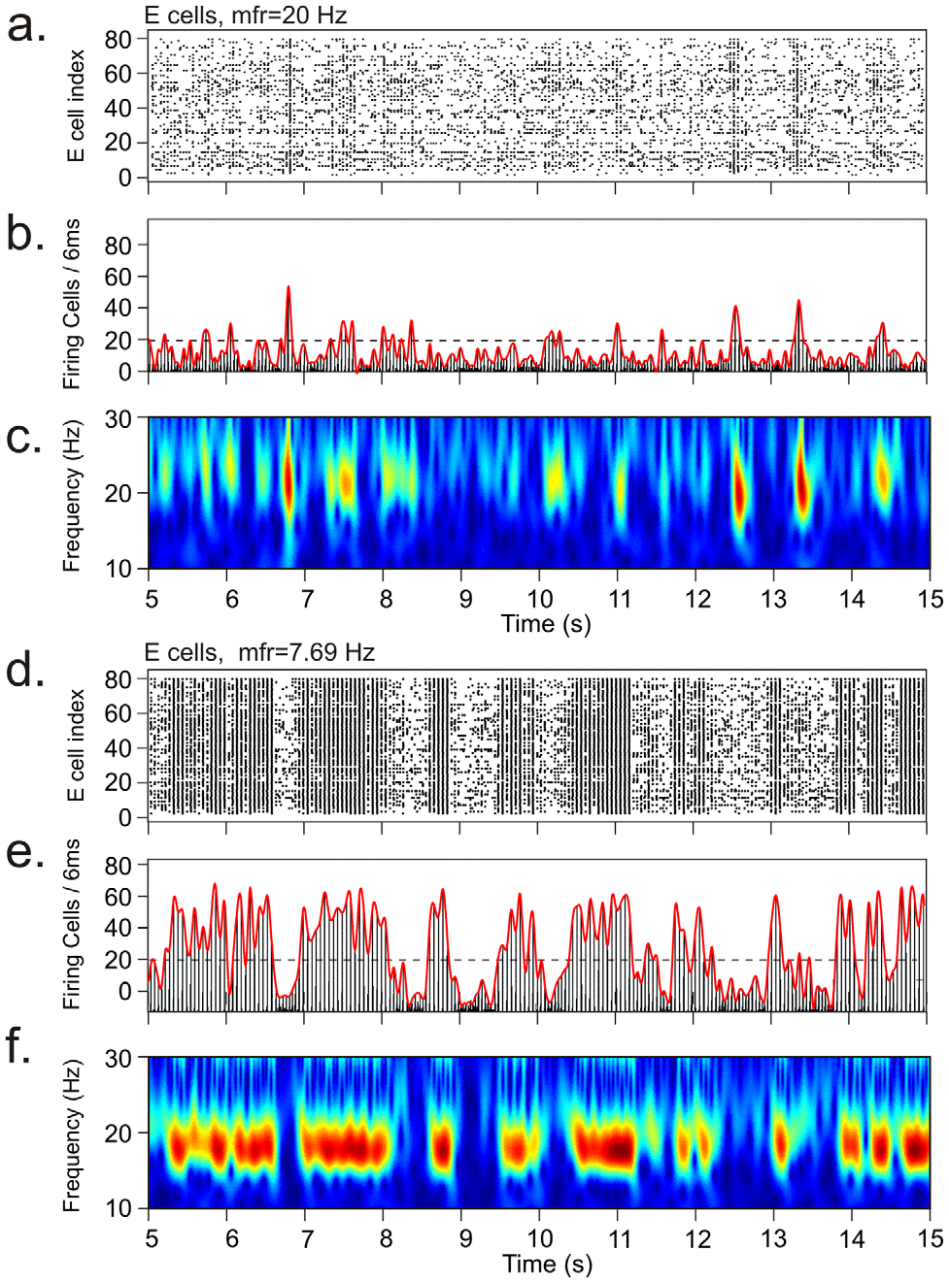
Alternating episodes of high- and low-amplitude oscillations for two different values of AP frequency. Raster diagrams of cell firing (a, d), firing-rate histograms with interpolated spline polynomials (b, e), and wavelet transforms of the firing-rate histograms (c, f) for the excitatory population for AP frequency is 20 Hz (a–c) and 7.69 Hz (d–f) in the minimal stimulation protocol. For an AP frequency of 20 Hz, the HAEs were much shorter and had a lower amplitude than for 7.69 Hz. The dashed horizontal line is the HAE threshold.

**Figure 8:**
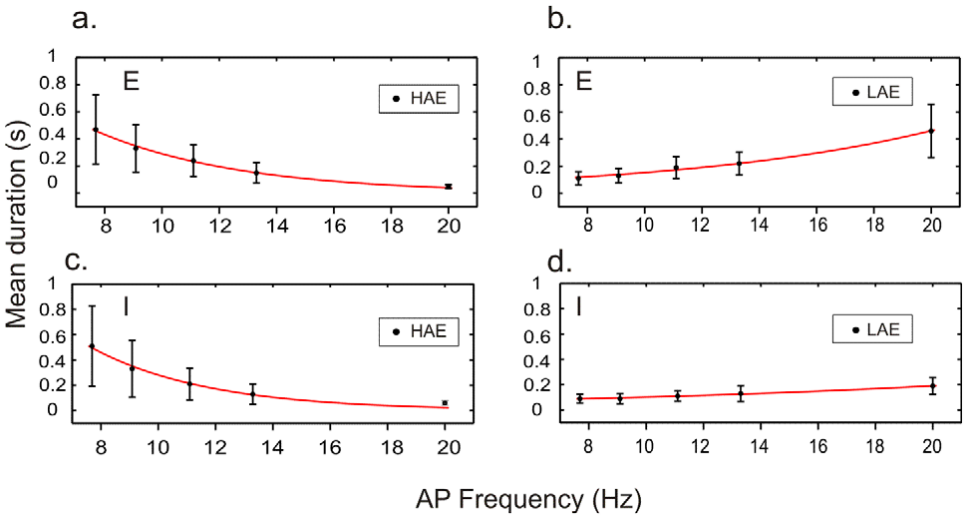
The higher the AP frequency, the shorter the mean HAE duration and the longer the mean LAE duration. Mean HAE and LAE durations (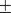
SEM) in the excitatory population (a, b) and the inhibitory population (c, d) for different values of AP frequency. Red lines, exponential fits.

To study the impact of AP firing frequency, we fixed AP randomness at 1 and varied the AP frequency between 7.69 and 20 Hz. Examples of network activity for two different values of AP firing frequency (7.69 and 20 Hz) are shown in Fig. 7. The mean HAE duration decreased and the mean LAE duration increased with increasing AP frequency, both in the excitatory population (Fig. 8a, b) and in the inhibitory population (Fig. 8c, d). Fig. S6 shows the distributions of HAE and LAE durations for different values of AP frequency.

### HAE-LAE alternations are caused by the interference of AP input with the ongoing oscillation

**Figure 9:**
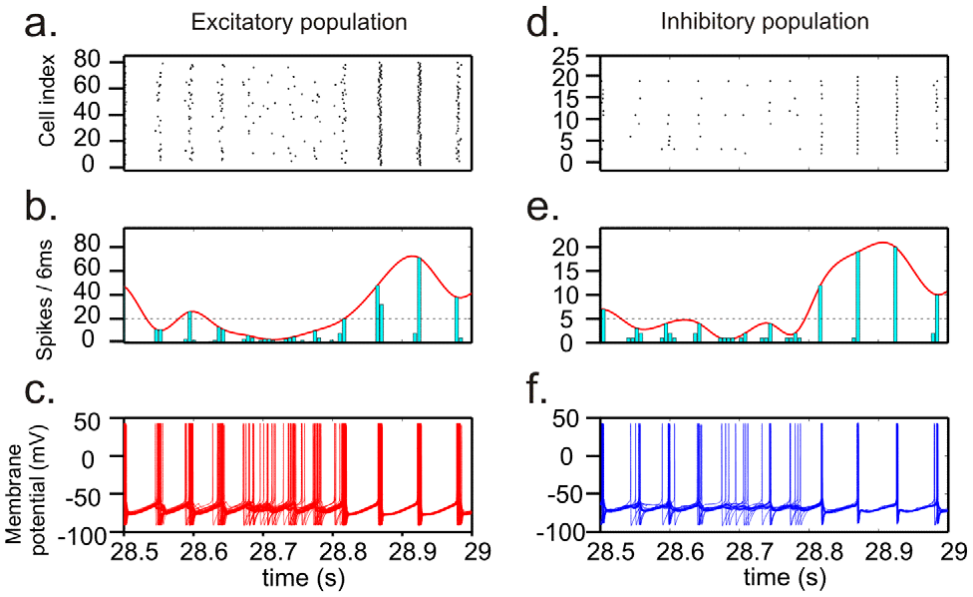
During a LAE, for both the excitatory and the inhibitory population, cell firing is less synchronous. This is revealed by the spread of activity over more time bins and the diminished overlap in membrane potential traces. In addition, fewer cells are firing during a LAE. Shown are the raster diagram of cell firing (a, d), the firing rate histogram with the spline polynomial (b, e), and the cell membrane potentials (c, f) of and interval of activity from Fig. 3. Horizontal dashed line, HAE threshold.

To elucidate the mechanism underlying HAE-LAE alternations, we first studied in more detail intervals of activity where transitions between HAE and LAE took place (Fig. 9). During a high-amplitude episode, cells fired in strong synchrony, resulting in high amplitudes of the firing rate histograms (high power in the wavelet map). During a low-amplitude episode, the cells fired less synchronously, as revealed by the spread of activity over more time bins. Less synchronous firing also implies that cells received synaptic input from other cells in the network less synchronously. Because simultaneous excitatory input from two or more firing cells was needed to generate an action potential, also the total number of cells firing during a LAE was often lower than during a HAE.

**Figure 10:**
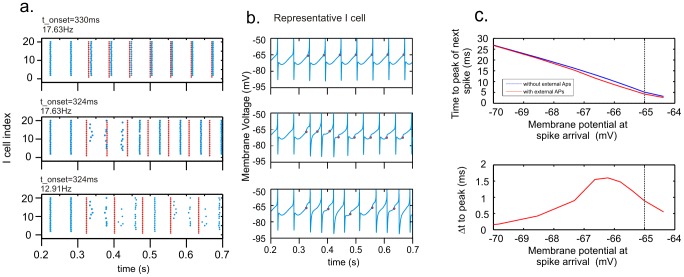
APs can disrupt synchrony among I cells, causing a LAE. (a) Red, external spikes (AP). Blue, inhibitory spikes. (top) AP frequency was the same as the frequency of the ongoing oscillation (17.63 Hz). If the first AP was delivered (at t_onset_ = 330 ms) when the membrane potential of the I cells was close to the firing threshold (between 0 and 0.7 mV), I cell firing was slightly advanced, but cells kept firing in synchrony. (middle panel) If the first APs was delivered when the membrane potential of the I cells was further below firing threshold (between 1 and 1.5 mV), I cell firing was reset and temporarily lost synchrony. (bottom) If AP frequency was lower than the frequency of the ongoing oscillation, the likelihood of APs resetting I cell firing increased, generating HAE-to-LAE transitions. (b) The firing pattern of a representative I cell for the different cases in (a). (c) The APs advanced the firing of the I cells compared with the expected firing dictated by the ongoing oscillation. The advancement depended on the cell's membrane potential at the time of AP arrival. The vertical line indicates the firing threshold.

Since HAE-LAE alternations also occurred with completely regular AP input (*rand* = 0) to the inhibitory cells, we further explored how AP input reduced synchronous firing by considering three specific cases with no randomness in the AP input (Fig. 10). The cases differed with respect to AP frequency and the time at which the APs were applied relative to the firing of the I cells.

In the first case (Fig. 10a, top), AP frequency was the same as the frequency of the ongoing oscillation, and APs were applied when the membrane potential of the I cells was just below (between 0 and 0.7 mV) the firing threshold. As a result, the I cells started firing slightly earlier, but network activity did not lose synchrony.

In the second case (Fig. 10a, middle), APs were applied at the same frequency as before but at times when the membrane potential was further below (between 1 and 1.5 mV) the firing threshold. The I cells advanced their firing times, but to a different extent, so that synchrony was lost.

Fig. 10c shows that the advancement in firing time depended on the cell's membrane potential at the time of AP arrival. When an AP arrived when the cell's membrane potential was close to the firing threshold or far below it, the AP had a relatively small effect on the timing of the subsequent spike.

In the third case (Fig. 10a, bottom), AP frequency was different from the frequency of the ongoing oscillation. During the course of the simulation, the APs arrived at different points in the oscillation cycle of the I cells and thus at different membrane potentials, resulting in varying levels of advancement in firing time and of synchrony.

In conclusion, HAE-LAE alternations are generated by the interference of the AP input with the ongoing network oscillations. The AP input to the I cells disrupts the synchrony of firing among the I cells (and consequently also among the E cells). As a result of the increased temporal spread of activity, the oscillation amplitude, defined as the number of firing cells during a 6 ms time interval, decreases and a LAE commences. After a variable period of time, the interactions between the excitatory and inhibitory cells are able to drive the network back to synchrony, leading to a HAE. Because the desynchronization effect of the AP input continually competes with the tendency of the excitatory and inhibitory connections to drive the network to synchrony [48], LAEs alternate with HAEs.

The stronger the disruption caused by the AP input, the more likely it is that network activity becomes desynchronized and that there is a switch from a HAE to a LAE. Consequently, the higher the AP frequency, the shorter the mean HAE duration will be (Fig. 8). Without any disruption, i.e., in the absence of AP input to the inhibitory population, the network will remain in a HAE for the duration of the entire simulation (Fig. S2). The disruptive effect of the AP input is also larger for higher randomness of the AP train, as different cells may receive AP input at different times and thus at different levels of their membrane potential, leading to different advances in their firing times and a higher likelihood that synchrony is lost. Consequently, the higher the AP randomness, the shorter the mean HAE duration will be (Fig. 6).

### The model reproduces HAE distributions observed in PFC

**Figure 11:**
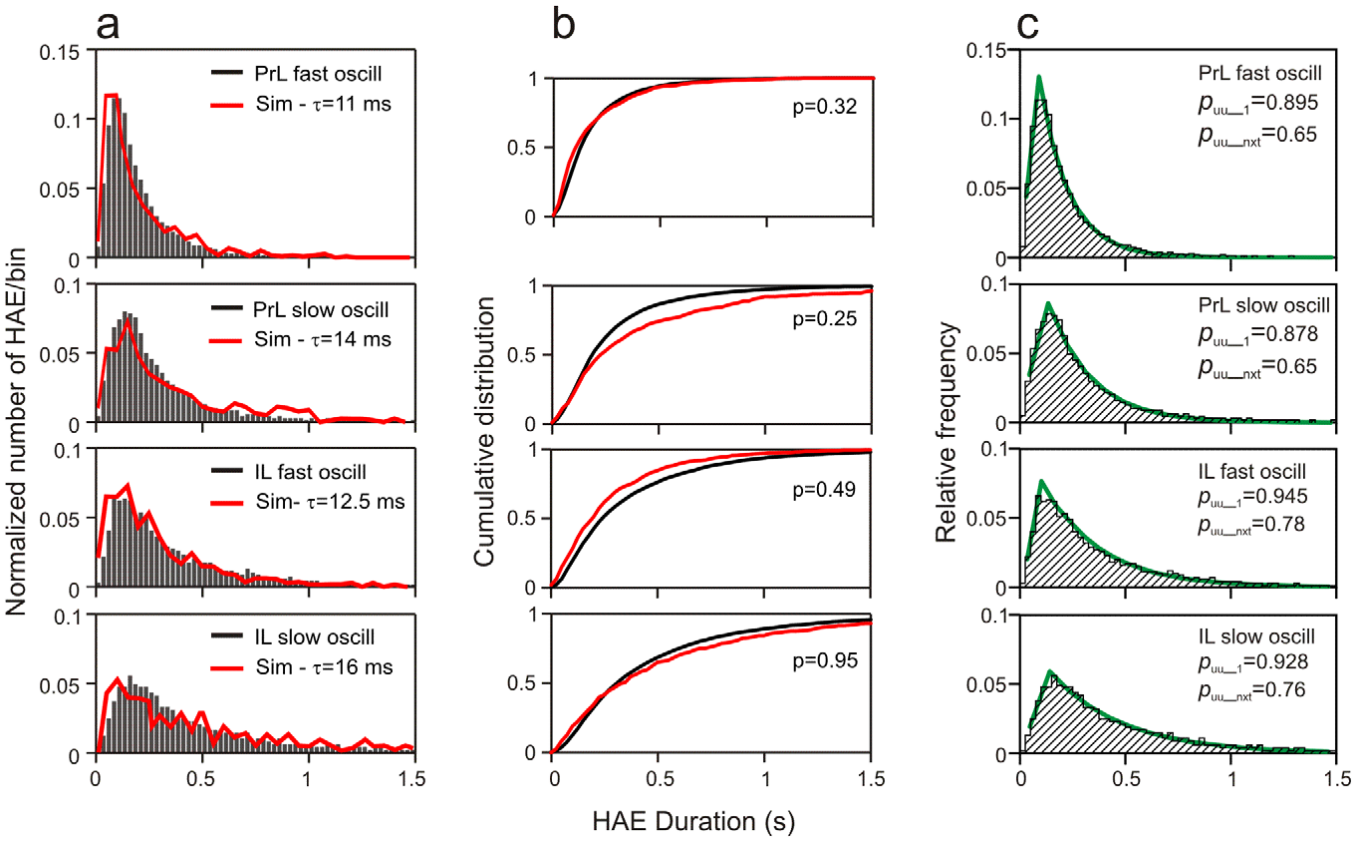
The distributions of HAE duration in the model match those observed for carbachol-induced oscillations in rat prefrontal cortex. (a) The model distributions (red lines) in the excitatory population and the empirical distributions (histograms) observed in the prelimbic (PrL) and infralimbic (IL) regions of the prefrontal cortex [52]. In each region of the PFC, both fast and slow oscillations occurred, which both exhibited HAE-LAE alternations. The oscillation frequency in the model was adjusted by changing the IPSC decay time τ. The distributions were normalized by dividing the number of HAEs within a given bin by the total number of HAEs in the distribution. (b) The cumulative distributions of the model data (red lines) and the empirical data (black lines). The model distributions are not significantly different (Kolmogorov-Smirnov test) from the empirical distributions. (c) The distributions generated by a Markov process (green line) accurately describe the empirical distributions (histograms). Parameter 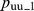 is the probability that the first oscillation cycle with high amplitude (upstate) in a HAE is followed by an upstate; 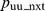 is the probability that an upstate in the rest of the HAE is followed by an upstate. See further main text.

We compared the distributions of HAE durations from our simulations with those observed in the prelimbic (PrL) and infralimbic (IL) regions of the PFC in rat slices [32], [52] (Fig. 11a, b). In both prelimbic and infralimbic areas, fast and slow oscillations occurred, referred to as PrL/fast (16.6±1.0 Hz), PrL/slow (11.2±0.5 Hz), IL/fast (14.7±0.7 Hz) and IL/slow (10.6±0.5 Hz), which all exhibited HAE-LAE alternation. To match the oscillation frequencies in the model with those in the PFC, we adjusted the decay time of the GABA-ergic synapses (Table S1). This resulted in HAE distributions that were already very similar to the experimental distributions with respect to their shapes. To further optimize the fit between model and experimental distributions, especially with respect to their means, we also adjusted CDC and AP frequency. To compare the experimental and model distributions of HAE durations, we used a two-sided Kolmogorov-Smirnov test (Fig. 11b). In all four cases, there were no significant differences between the experimental distributions and the model-generated distributions.

To get more insight into what the shapes of the experimental and model HAE distributions tell us about the transitions between synchronous and desynchronous activity, we analyzed the experimental data assuming a Markov process for the succession of high- and low-amplitude cycles. A single cycle of the ongoing oscillation is either in an upstate, when it has synchronized activity and consequently a high amplitude (and thus is part of a HAE), or in a downstate, when it has desynchronized activity and consequently a low amplitude (and thus is part of a LAE). Let 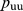 and 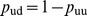 denote the transition probabilities from an upstate to an upstate and from an upstate to a downstate, respectively. Similarly, let 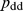 and 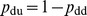 denote the transition probabilities from a downstate to a downstate and from a downstate to an upstate, respectively. The probability of a HAE consisting of 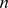 cycles in an upstate, preceded and followed by a cycle in a downstate, is then proportional to 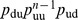, which is an exponentially decreasing function of 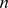. However, the distributions in Fig. 11a show that HAE duration probability has a clear modus, with much lower probabilities for short HAEs, i.e., HAEs with a short sequence of upstates. Apparently, the transition probability of a cycle depends on its position in a HAE. To be able to match the shape of the experimental HAE distributions, we found that it was sufficient to assume that the probability 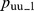 that the first upstate in a HAE is followed by an upstate is higher than the probability 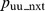 that an upstate in the rest of the HAE is followed by an upstate. Fig. 11c shows that the distributions generated by a Markov process with such transition probabilities accurately describe the experimental HAE distributions. Because of the strong similarity between model and experimental data, such probabilities are thus also required to account for the model data. In conclusion, the succession of high-amplitude cycles (i.e., synchronized activity) and low-amplitude cycles (i.e., desynchronized activity) is a random process governed by a slightly modified Markov process. The Markov analysis revealed that a cycle with synchronized activity that directly follows upon a cycle with desynchronized activity is apparently less vulnerable to become desynchronized again in the next cycle.

### The model reproduces the effect of carbachol on HAE duration in the hippocampus

**Figure 12:**
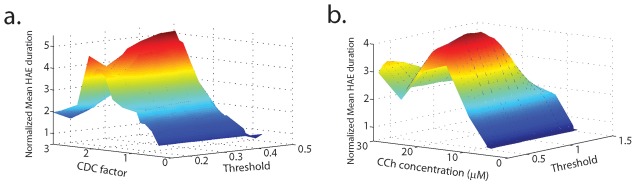
In the model, as in mouse hippocampus, the mean duration of high-amplitude episodes varies in a bell-shaped manner with CDC input or carbachol concentration. (a) For different values of the HAE threshold, the mean duration of the HAEs in the model for the excitatory population as a function of CDC input. The CDC input represents cholinergic input. (b) The bell-shaped relationship between cholinergic input and mean HAE duration was confirmed experimentally by using the cholinergic agonist carbachol (CCh) applied to mouse hippocampal slices. A HAE was defined as an episode in which the amplitude envelope of the bandpass-filtered oscillations was above 0.5*the mean oscillation amplitude [42]. For a given HAE threshold, the mean HAE durations were normalized by dividing by the mean HAE duration obtained for the lowest CCh concentration different from zero. Fig. b modified from [42].

The CDC input to the model network represents cholinergic input and in cortical slice experiments is often provided by applying the cholinergic agonist carbachol [9], [60]. To study the impact of CDC input on HAE duration, we varied the strength of CDC by multiplying the standard value (of both the excitatory and the inhibitory population; see Methods) by a factor ranging from 0.3 to 3. To ensure that the results were not critically dependent on the choice of the HAE threshold, we analyzed the network activity using six different values of the HAE threshold. The results (Fig. 12a) show that HAE duration increased with increasing CDC input and, interestingly, decreased when CDC input was further increased. This bell-shaped relationship between CDC input and mean HAE duration held for all values of the HAE threshold.

With low CDC (CDC factor <1.2), which raised the cells' membrane potential marginally above resting level, there was no clear oscillatory activity. The overall activity in the network was low, and cells fired non-synchronously, as a result of the randomness of the AP drive. With higher CDC (1.2<CDC factor<3), clear oscillatory activity was seen with HAE-LAE alternations. In this range of CDC values, which markedly raised the cells' membrane potential above resting level, HAE duration first increased with increasing CDC. However, when CDC was further increased, the CDC tended to bring the membrane potential above firing threshold, so that the firing directly induced by the synaptic interactions between the cells in the network became less important, resulting in less synchronous activity and decreased HAE durations.

Our model results are in agreement with results from experiments in which oscillations in mouse hippocampal brain slices were induced by applying different concentrations of carbachol [42]. These oscillations had a frequency of about 18 Hz and fluctuated in amplitude. As in the model, a bell-shape relationship was found between carbachol concentration and mean HAE duration (Fig. 12b). HAE duration increased with increasing carbachol concentration (1–15 µM) and decreased when carbachol concentration was further increased (15–25 µM). In [42] the authors tested for a difference in median HAE duration with one-tailed paired permutation tests between 5 µM and 15 µM, and 15 µM and 20 µM. Indeed, HAE duration was significantly higher at 15 µM compared with 5 µM and 20 µM (Friedman test, p<0.05, n>17, binomial corrected). The tendency for oscillations to have long-lasting HAEs at intermediate levels of carbachol was present for all HAE thresholds.

## Discussion

Irregular fluctuations in the amplitudes of ongoing oscillations have been observed in many frequency bands and brain regions, but the mechanisms by which they are generated are unknown. We showed here that a generic model network consisting of interconnected excitatory and inhibitory cells, which were driven by action potentials (activating excitatory synapses) and current input (cholinergic stimulation) from areas external to the network, generated oscillatory activity in the alpha/beta frequency band in which high-amplitude episodes (HAEs) alternated irregularly with low-amplitude episodes (LAEs). The minimal condition for these HAE-LAE alternations to arise was external action potential input (representing, e.g., corticocortical or thalamocortical input) onto the inhibitory cells combined with current input to both the excitatory and the inhibitory cells.

The external action potential (AP) input interferes with the ongoing network oscillations generated by the reciprocal interactions between excitatory and inhibitory cells. The APs advance the firing time of the inhibitory cells to an extent that depends on the cell's membrane potential at the time of AP arrival. As a consequence, cells experience a variable degree of advance in their firing times, leading to a reduction in firing synchrony. Since oscillation amplitude is proportional to the number of simultaneously firing cells, reduced synchrony gives rise to a decrease in oscillation amplitude and the start of a LAE. The desynchronization effect of the AP input competes with the tendency of the interacting excitatory and inhibitory cells to drive the network back to synchrony, so that LAEs alternate with HAEs. Note that randomness in the AP train is not per se required for the APs to be able to desynchronize network activity (see Fig. 5d–f).

The occurrence of alternating episodes of high- and low-amplitude oscillations was robust to changes in model properties, including the total number of cells in the network, the ratio of excitatory to inhibitory cell numbers, the connectivity structure of the network as determined by the connection probabilities between excitatory and inhibitory cells, the decay time constants of the synaptic conductances, the duration of the synaptic delay, and cellular properties such as the channel conductances underlying the generation of action potentials. In particular, increasing the total number of cells in the network ten-fold, while maintaining the values of all other parameters and the proportion of excitatory and inhibitory cells, did not affect the results. Changing the ratio of excitatory to inhibitory cell numbers from 80%–20% to 70%–30% or 90%–10% affected the durations of LAEs and HAEs, but not the presence of fluctuations in oscillation amplitude as such. The qualitative results were also largely insensitive to different choices of connection probabilities, as long as they supported the generation of oscillations. The IPSC decay constant affected the frequency of the oscillations, with lower frequencies as the IPSC decay constant increased, but did not qualitatively influence the occurrence of LAEs and HAEs, as shown in Fig. 11, where the IPSC decay time constant was varied between 11–16 ms. The synaptic delay in our standard network was 1 ms; only for much larger delays (15–20 ms), with all the other parameter values unchanged, did the network fail to show HAE-LAE alternations. Altering the conductances of the Na^+^, K^+^ and leakage channels by about 30% had impact only on the shape of the action potentials but not on the network oscillations and the amplitude fluctuations. In conclusion, our results are not dependent on a particular choice of parameter values and may therefore be valid for a wide range of brain networks.

It is important to note that the oscillations in our model are not generated by a stochastic resonance mechanism. The ongoing network oscillations are generated by the interacting excitatory and inhibitory cells, whereas the noisy external drive tends to perturb the oscillations. With stochastic resonance, in contrast, it is the noisy drive that induces oscillations, when the system resonates at a particular noise level [61].

External action potential input applied onto excitatory cells was not capable of reducing synchronous firing and thus inducing low-amplitude episodes. Even if the strength of the synapse upon which the external action potential input impinges was increased up to 3–4 times, the external action potentials were not able to desynchronize the oscillations. The inhibitory cells, rather than the excitatory cells, are responsible for synchronizing cell firing and for setting the rhythm of the network [48]. The oscillations are basically generated through periodic silencing of the network by the inhibitory cells, with the IPSC decay time determining the frequency of the oscillations.

The distributions of high-amplitude episode (HAE) durations in the model matched those observed experimentally in different PFC areas. To obtain the same oscillation frequencies in the model as in the experimental recordings, we adjusted IPSC decay time, as IPSC kinetics have been shown to modulate oscillation frequency [2], [32]. To further optimize the fit between model and experimental HAE distributions, we also adjusted the constant depolarizing current (CDC) input and the frequency of the train of external action potentials (AP). Different values of CDC and AP frequency are to be expected as different PFC areas may receive different external input.

With increasing randomness or frequency of the external spike train, the mean HAE duration became shorter and the mean LAE duration longer. This prediction of the model can be tested experimentally in cortical slices cultured on multi-electrode arrays (MEAs). Since MEAs allow not only the recording of field potentials but also the delivery of electrical signals, with full control over the randomness and frequency of the stimulation, it can be tested whether HAE duration is influenced by these stimulation parameters in the same manner as observed in the model.

The mean HAE duration increased with increasing CDC input (representing cholinergic stimulation), but decreased when the current input was further increased. This bell-shaped dependence was also observed in hippocampal slice cultures in which the duration of HAEs was measured for different concentrations of carbachol applied [42].

Although the spatial scales of the neuronal networks considered here are different from those of the networks measured with EEG, also EEG measurements reveal similar fluctuations in oscillation amplitude [40], with periods of high amplitude alternating with periods of low amplitude. Interestingly, the mean duration of episodes of high-amplitude oscillations is similar across many systems and in agreement with our model outcomes: acute slices of rat prefrontal cortex (PrL fast: 192 ms; IL slow: 476 ms; see Results) [32], alpha/beta oscillations in acute hippocampal slices (450 ms) [42], theta/alpha oscillations in human EEG (546 ms) [41], and alpha oscillations in human EEG (518–555 ms) [28]. Also, the mean HAE duration expressed in number of oscillation cycles is in all systems mostly in the range of 3–7 cycles. The duration of single HAEs varies strongly, both in the model and in the cortex (Fig. 11a), and can be accurately described by a slightly modified Markov process (Fig. 11c). If slow hyperpolarizing channels, such as slow calcium-activated potassium channels, were responsible for alternating HAEs and LAEs, one would expect that the durations of HAEs and LAEs would be much less variable, being determined by the kinetics of the channels.

In model studies, Börgers et al. [7], [8] found that hyperpolarizing adaptation currents can change the state of the network from asynchrony to weak gamma rhythmicity. They also described a parameter regime in which the network toggles between a state of gamma rhythmicity and a state in which the E-cells are suppressed by asynchronous activity of the I-cells. This dynamics is somewhat reminiscent of the dynamics that we have found here, except that in our case most E-cells during a LAE keep firing but with reduced synchrony. Another model study [62] showed that in networks consisting of only inhibitory neurons, synaptic noise or heterogeneity in the applied current can reduce the degree of synchrony; however, no alternating episodes of high synchrony (i.e., HAEs) and low synchrony (i.e., LAEs) were reported. In model simulations, Tiesinga et. al. [9], [63] studied carbachol-induced transitions between oscillations of different frequencies. These studies were, however, not concerned with amplitude variability or transitions between high- and low-amplitude episodes. In [64], synchrony and stability, but no HAE-LAE alternations, were investigated in general pulse-coupled oscillators. Unlike these oscillators, the cells in our network are excitatory or inhibitory, are not connected all-to-all, and do not fire when uncoupled but require synaptic input from the other cells in the network to become activated. In a corticothalamic model, Freyer et al. [28] found that noisy synaptic inputs into thalamic neurons elicit bursts between low- and high-amplitude oscillations if the system is near a particular type of dynamic instability. The model describes the mean field dynamics of populations of excitatory and inhibitory neurons in the cortex as they interact with neurons in the thalamus. Thus no individual neurons are considered, so that in their model, in contrast to our model, firing synchrony between cells and the impact of noisy input on synchrony and oscillation amplitude cannot be investigated. Their model also needs a rather complex synaptic noise term, containing both an activity-dependent, multiplicative component and a purely additive component. Moreover, since alternating episodes of high- and low-amplitude oscillations also occur in isolated cortical slices [3], the mechanism by which they are generated cannot depend on thalamic input.

Our study suggests that amplitude fluctuations may be a general property of oscillatory neuronal networks that can arise through background input from areas external to the network. Episodes of high-amplitude oscillations reflect periods of synchronized firing. Since synchronized firing between cells is important for correlation-based Hebbian learning, HAEs provide favorable conditions for synaptic strength modification. As we have shown here, external input to a network can modulate the duration of HAEs and thus may influence periods of learning and memory formation. Also, the external input changes the relative times of the postsynaptic spikes in the network and could thereby affect the degree and even sign of spike-timing synaptic plasticity (STDP). Indeed, in the hippocampus it has been found that afferent input can, depending on the timing of the stimulation, either advance or delay the postsynaptic spike and thus determine whether synaptic potentiation or depression takes place [65].

## Supporting Information

Figure S1 **Schematic diagram of the network.** The cells of the inhibitory (I) population projected among each other and to the cells of the excitatory (E) population. The cells of the excitatory population projected among each other and to the cells of the inhibitory population. Both excitatory and inhibitory cells also received external input in the form of a constant depolarizing current (CDC) and a train of action potentials (AP) that activate an excitatory synapse.
(TIF)

Figure S2 **HAE-LAE transitions did not occur without AP input to the inhibitory cells.** In this example, both the excitatory and the inhibitory population received CDC input, but only the excitatory population received AP input (see also Fig. 4g). For the excitatory cells, the figure shows the raster diagram of cell firing (a), the firing-rate histogram with the spline polynomial (b), and the wavelet transform of the firing-rate histogram (c). Panels (d) and (e) depict a zoomed-in period of (a) and (b), respectively, as indicated by the red lines below the x-axes. The apparent amplitude variation is a consequence of the binning procedure (see Methods), and does not reflect a genuine alteration in activity level. The discrete binning may cause a single event of maximal network-wide firing to become divided over two consecutive bins. This will then show up as a lower amplitude of the oscillation. However, the quantification of HAE-LAE alternation was not affected, because the apparent drop in amplitude never fell below the HAE threshold.
(TIF)

Figure S3 **The effect of AP randomness (rand) and AP frequency (mfr) on the distribution of oscillation amplitudes in the excitatory population for the minimal stimulation protocol.** The red dashed line is the HAE threshold. Low AP frequencies (0.1–4 Hz) could hardly affect the ongoing oscillation (which had a frequency of about 18 Hz), so that most amplitudes remained supra-threshold. High AP frequencies (35–125 Hz) disturbed the ongoing oscillation so much that firing synchrony was lost, resulting in low, sub-threshold amplitudes. For intermediate AP frequencies (between 4 and 35 Hz), the amplitude distribution contained both supra- and sub-threshold parts. The supra-threshold fraction of the distribution tended to increase with lower AP randomness.
(TIF)

Figure S4 **Distributions of HAEs and LAEs in the excitatory population for different values of AP randomness in the minimal stimulation protocol.** The p-values indicate the similarity of each distribution to the reference distribution with rand = 1 (Kolmogorov-Smirnov test). Except for rand = 0.9, there were significant differences with the reference distributions. For rand = 0.1 (*), there is a time bin outside the potted range, at 12.86 s, with two episodes.
(TIF)

Figure S5
**Influence of mode of AP delivery on HAE duration distributions.** For the excitatory population, the three columns show, from left to right, the firing times of the external spikes onto the inhibitory cells, the interspike interval (isi) distribution of the external spikes, and the distribution of HAE durations. For the HAE durations, the mean
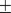
std and p-values (Kolmogorov-Smirnov test, testing the distribution against that obtained with rand = 1) are given. In the first four rows, all inhibitory cells received simultaneously (in phase) their first external spike at 80 ms after the onset of the simulation. Because of the randomness in assigning the subsequent spikes, the firing times of the external spikes quickly ran out of phase, except for rand = 0. In the bottom row (out of phase), for rand = 0, the first external spike was uniformly randomized between 0–80 ms. For all modes of AP delivery, including rand = 0, HAEs and LAEs occurred (see also Fig. 5d–f).(TIF)

Figure S6 **Distributions of HAEs and LAEs in the excitatory population for different values of AP frequency (mfr) in the minimal stimulation protocol.** The p-values indicate the similarity of each distribution to the reference distribution with AP frequency 11.11 Hz (Kolmogorov-Smirnov test). Only the distributions for AP frequencies 20 and 7.69 Hz differed significantly from the ones of the reference situation.
(TIF)

Table S1 **Oscillation frequency and mean and median HAE duration observed in the model (excitatory population) and in the prelimbic (PrL) and infralimbic (IL) regions of the prefrontal cortex (PFC).** Also listed are the values of CDC factor, IPSC decay time and AP frequency used in the model to fit the oscillation frequency and HAE duration distributions in the PFC. In each region of the PFC, both fast and slow oscillations occurred.
(DOC)
