# Supplementary figures and images for: External Drive to Inhibitory Cells Induces Alternating Episodes of High- and Low-Amplitude Oscillations
Source: PLoS Comput Biol. 2012 Aug 30;8(8):e1002666. doi: 10.1371/journal.pcbi.1002666 (PMC3431298; doi:10.1371/journal.pcbi.1002666)

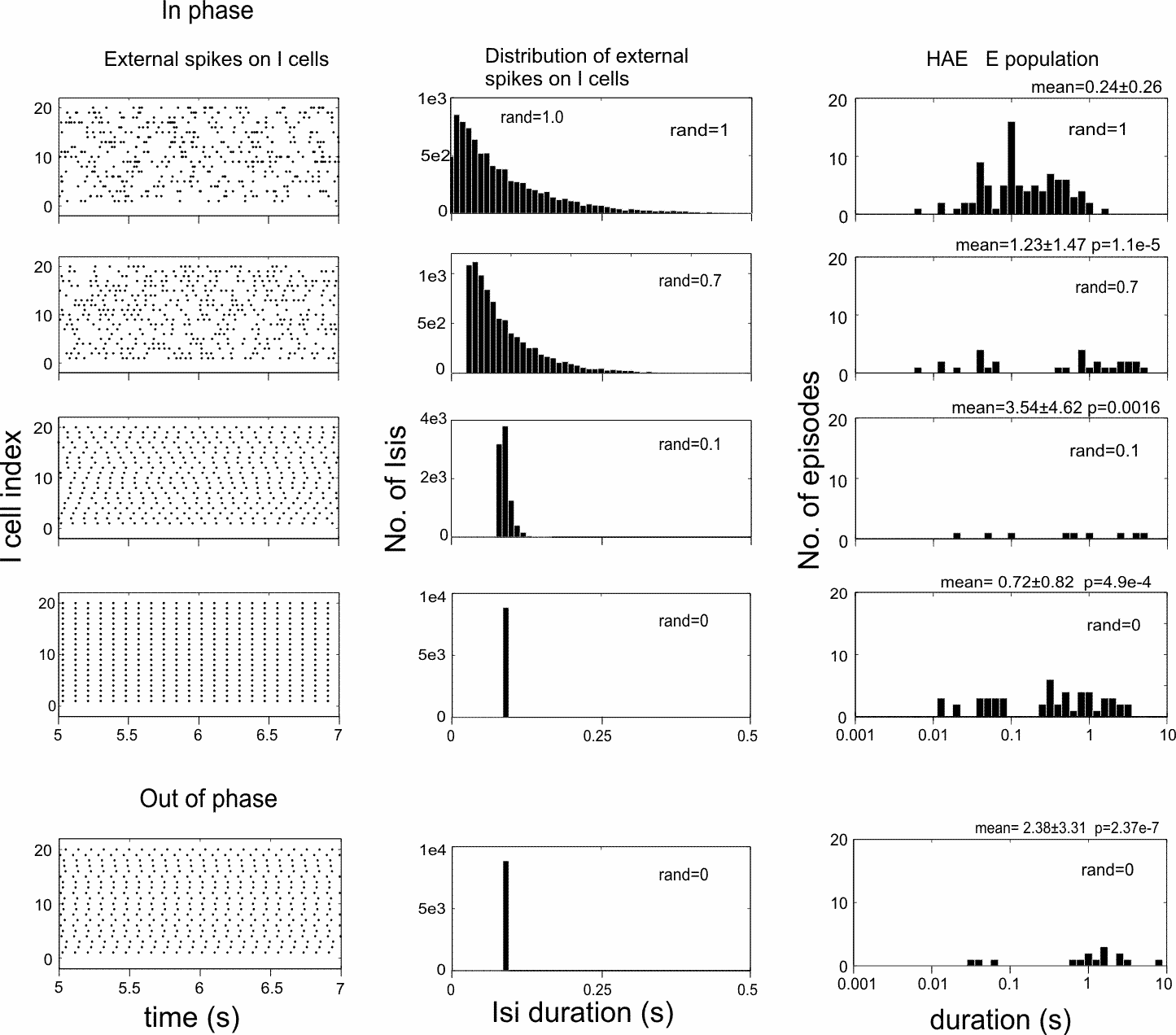

Supplement: Figure S5 — Influence of mode of AP delivery on HAE duration distributions. For the excitatory population, the three columns show, from left to right, the firing times of the external spikes onto the inhibitory cells, the interspike interval (isi) distribution of the external spikes, and the distribution of HAE durations. For the HAE durations, the meanstd and p-values (Kolmogorov-Smirnov test, testing the distribution against that obtained with rand = 1) are given. In the first four rows, all inhibitory cells received simultaneously (in phase) their first external spike at 80 ms after the onset of the simulation. Because of the randomness in assigning the subsequent spikes, the firing times of the external spikes quickly ran out of phase, except for rand = 0. In the bottom row (out of phase), for rand = 0, the first external spike was uniformly randomized between 0–80 ms. For all modes of AP delivery, including rand = 0, HAEs and LAEs occurred (see also Fig. 5d–f). (TIF) [file pcbi.1002666.s005.tif]
