# Supplementary material for: External Drive to Inhibitory Cells Induces Alternating Episodes of High- and Low-Amplitude Oscillations
Source: PLoS Comput Biol. 2012 Aug 30;8(8):e1002666. doi: 10.1371/journal.pcbi.1002666 (PMC3431298; doi:10.1371/journal.pcbi.1002666)
Supplement: Table S1 — Oscillation frequency and mean and median HAE duration observed in the model (excitatory population) and in the prelimbic (PrL) and infralimbic (IL) regions of the prefrontal cortex (PFC). Also listed are the values of CDC factor, IPSC decay time and AP frequency used in the model to fit the oscillation frequency and HAE duration distributions in the PFC. In each region of the PFC, both fast and slow oscillations occurred. (DOC) [file pcbi.1002666.s007.doc]

| PFC area/  oscill. type | Oscillation frequency (Hz) | Mean HAE (ms) | Median  HAE (ms) | CDC  factor | IPSC  decay (ms) | AP frequency (Hz) |
| --- | --- | --- | --- | --- | --- | --- |
| PrL/slow | Exp:  11.2±0.5  Model:  11.39 | 284  408 | 200  240 | --  0.52 | --  14 | --  4.54 |
| PrL/fast | Exp:  16.6±1.0  Model:  16.3 | 192  189 | 150  114 | --  0.87 | --  11 | --  10.53 |
| IL/slow | Exp:  10.6±0.5 Model:  10.42 | 476  366.6 | 325  228 | --  0.55 | --  16 | --  2.63 |
| IL/fast | Exp:  14.7±0.7  Model:  14.81 | 381.41  396.7 | 250  204 | --  0.9 | --  12.5 | --  7.14 |
